# Head movement kinematics are differentially altered for extended versus short duration gait exercises in individuals with vestibular loss

**DOI:** 10.1038/s41598-023-42441-2

**Published:** 2023-09-27

**Authors:** Jennifer L. Millar, Omid A. Zobeiri, Wagner H. Souza, Michael C. Schubert, Kathleen E. Cullen

**Affiliations:** 1 Department of Physical Medicine and Rehabilitation, Johns Hopkins University School of Medicine, Baltimore, MD USA; 2 Department of Biomedical Engineering, Johns Hopkins University, Baltimore, MD USA; 3 Department of Biomedical Engineering, McGill University, Montreal, QC Canada; 4 Department of Otolaryngology-Head and Neck Surgery, Johns Hopkins University School of Medicine, 720 Rutland Ave, Traylor 504, Baltimore, MD 21205-2109 USA; 5 Department of Neuroscience, Johns Hopkins University School of Medicine, Baltimore, USA; 6 Kavli Neuroscience Discovery Institute, Johns Hopkins University, Baltimore, MD USA

**Keywords:** Neuroscience, Engineering

## Abstract

Head kinematics are altered in individuals with vestibular schwannoma (VS) during short duration gait tasks [i.e., Functional Gait Assessment (FGA)], both before and after surgery, yet whether these differences extend to longer duration gait exercises is currently unknown. Here we examined the effects of vestibular loss and subsequent compensation on head kinematics in individuals with VS during gait exercises of relatively extended versus short duration (< 10 versus 30 s), compared to age-matched controls. Six-dimensional head movements were recorded during extended and short duration gait exercises before and then 6 weeks after sectioning of the involved vestibular nerve (vestibular neurectomy). Standard functional, physiological, and subjective clinical assessments were also performed at each time point. Kinematics were differentially altered in individuals with vestibular loss at both time points during extended versus short duration exercises. Range of motion was significantly reduced in extended tasks. In contrast, movement variability predominately differed for the short duration exercises. Overall, our results indicate that quantifying head kinematics during longer duration gait tasks can provide novel information about how VS individuals compensate for vestibular loss, and suggest that measurements of range of motion versus variability can provide information regarding the different strategies deployed to maintain functional locomotion.

## Introduction

The vestibular system detects the head’s six-dimensional motion and orientation relative to space to provide essential sensory information to the brainstem, cerebellum, and vestibular cortex during our everyday activities^1^. In healthy individuals, coordinated vestibular-motor pathways make essential contributions to the multisensory mechanisms required to maintain stable balance during our everyday activities (reviewed in Ref.^2^). For instance, to transition from a stable standing posture to locomotion, the brain switches between reflex and voluntary vestibular motor pathways^3^, in a coordinated, goal directed, manner consistent with optimal feedback theory^4,5^. The reduction or absence of peripheral vestibular input to these pathways increases the brain’s reliance on information from other sensory modalities (i.e., visual and somatosensory systems), which in turn contribute to long-term vestibular compensation (reviewed in Refs.^6–8^). However, such compensation is incomplete, resulting in individuals with vestibular loss requiring significantly more time to perform daily life activities compared to controls^9^.

Locomotion, the ability to walk from place to place, is an essential aspect of our daily living. To date, head kinematics have been studied in healthy individuals during locomotion, in conditions including treadmill walking^10–12^ as well as free overground walking^13–19^). Overall, these studies have led to the proposal that head stabilization, particularly in the pitch axis, is a vital feature of inertial guidance and postural control during locomotion^14^. To understand how loss of vestibular sensory input alters locomotion, several research studies have measured and quantified head kinematics in individuals with peripheral vestibular loss. Measurements have been made using magnetic search coils^12^, 3D video motion capture^13,14,20,21^, and most recently inertial measurement units (IMUs)^22–25^. Compared to controls, individuals with peripheral vestibular loss display marked changes in head movement variability, principally along the vertical axis, as well as time for task completion. Overall, these studies have contributed to the proposal that head stabilization is important for navigation and postural control. The use of IMUs to quantify kinematics in more than 2 dimensions is relatively novel and unexplored. Indeed to date, only two studies have measured 6-dimensional head kinematics in individuals with peripheral loss^23,24^. Both of these prior studies focused on performance during a traditional clinical gait measure, the Functional Gait Assessment (FGA), which comprises only short duration gait tasks (i.e., < 10 s). Thus, it remains unknown how 6-dimensional head kinematics are altered in such individuals during longer duration bouts of locomotion that are common in daily living.

Accordingly, via this exploratory study we measured and compared head kinematics during extended versus short duration gait tasks in individuals with vestibular loss pre and post vestibular deafferentation. Six-dimensional head kinematics were recorded in vestibular schwannoma participants, at pre-operative and 6 weeks post-operative time points, relative to healthy controls. Measurements were made using light weight IMU sensors during 7 30-s vestibular gait exercises detailed by the Clinical Practice Guidelines for vestibular rehabilitation in individuals with peripheral vestibular hypofunction^26^, as well as during the 10 standard tasks of the FGA (< 10 s in duration). Notably, 4 of these former tasks were extended versions of FGA tasks. We first tested whether head kinematics distinguished VS from healthy control participants in either the pre- and postoperative state during extended gait tasks. We then assessed whether there were significant differences in kinematics of these individuals during extended versus short duration tasks, relative to healthy controls. Finally, we investigated whether and how the quantification of head kinematic data during both extended and short duration gait tasks can be utilized to predict standard clinical, functional, or physiological outcomes in the VS pre and/or postoperative state relative to healthy controls.

## Methods

Subjects: 18 participants with vestibular schwannoma (VS) tumor were recruited and scheduled for surgical resection between February 2019 and January 2020. 9 VS participants (male = 9, mean 52 ± 15 years old, range 23–70 years old) were assessed before (mean = 8 ± 13 days) and 6 weeks after (35–45 days) surgery, (right VS tumor = 5, left = 4). We chose this 6-week post-operative time point to correspond when participants returned to Johns Hopkins for their scheduled post-operative follow up otology appointment. 9 aged matched healthy controls were also recruited (8 males and 1 female, mean 49.3 ± 15.0 years old, range 24–72 years old). Details of the VS participant demographic information as well as tumor size, laterality, and Koos score are provided in Supplementary Table 1. The pre-operative symptoms of the 9 VS participants were as follows: 78% complained of auditory impairment with 100% having documented sensorineural hearing loss via audiogram. Other pre operative symptoms included dizziness (44%), imbalance (44%), tinnitus (33%), pain (33%), headache (22%), ear fullness (22%), and facial motor involvement (22%). Participants performed the Functional Gait Assessment, a standard rehabilitation outcome measure that includes 10 dynamic postural stability tasks (~ 10 s each in duration), as well as 7 longer duration vestibular rehabilitation gait exercises (~ 30 s each) while wearing 6 IMUs (head, upper trunk, lower trunk (waist) right and left ankle, dominant hand). The kinematic and functional measures were captured simultaneously and included in the data collection, as well as physiological and subjective measures pertaining to participants symptoms and perceived level of function. This prospective study was approved by the Johns Hopkins Institutional Review Board and performed according to the institution’s guidelines for safe and ethical research in human subjects. Informed consent was obtained from all subjects and/or their legal guardian(s) prior to data collection.

### Clinical measures

#### Dynamic visual acuity (DVA)

DVA measures visual acuity during active head rotation as a proxy of the vestibular ocular reflex using a portable test developed and validated at our institution. DVA is a behavioral measure of the vestibular ocular reflex with active head rotation with visual fixation of an optotype. The DVA test comprises of a Samsung Galaxy Pro tablet (Seoul, South Korea) and a head mounted motion sensor (XSENS Technologies, Enschede, Netherlands). We used the DVA protocol as previously described by Millar et al.^27^. Participants performed the DVA test while seated, 2 m from the portable tablet. 10 optotypes (C D H K N O S R V Z) were randomly presented during 3 different conditions: (1) head stationary (static visual acuity—SVA) (2) sinusoidal head rotations with the optotype presented during active right head rotation and (3) active left head rotation. The scores were calculated in the logarithm of the minimal angle resolution (LogMAR). Possible LogMAR scores ranged from − 0.3 to 1.7 (Snellen equivalent of 20/10 to 20/800). Corrected DVA accounted for static visual acuity scores by subtracting SVA from DVA.

#### Timed up and go (TUG)

Participants were asked to stand from a seated position, walk 3 m, turn 180° and then return to a seated position. The TUG task was performed for 2 trials, with an ispilesional turn and contralesional turn, respectively. For healthy controls, the two trials equivalent to ipsilesional and contralesional turns were carried with a right turn and left turn, respectively. The TUG has excellent inter and intra-rater reliability in community dwelling elderly (ICC = 0.99)^28^. Higher TUG scores, > 11.1 s, correlate with reports of falls in individuals with vestibular dysfunction^29^.

#### Gait speed

The Ten Meter Walk Test (10MWT) was performed, measuring participants’ self-selected walking pace. The walking duration was timed over a 10-m distance. Participants started and stopped their walking distance both 2 m before and after the 10-m timed portion of the walk to ensure the acceleration and deceleration phases of locomotion were not included in the timed portion of the test. The average gait velocity (meters/second) was computed based on two consecutive trials.

#### Functional gait assessment (FGA)

**Figure 1:**
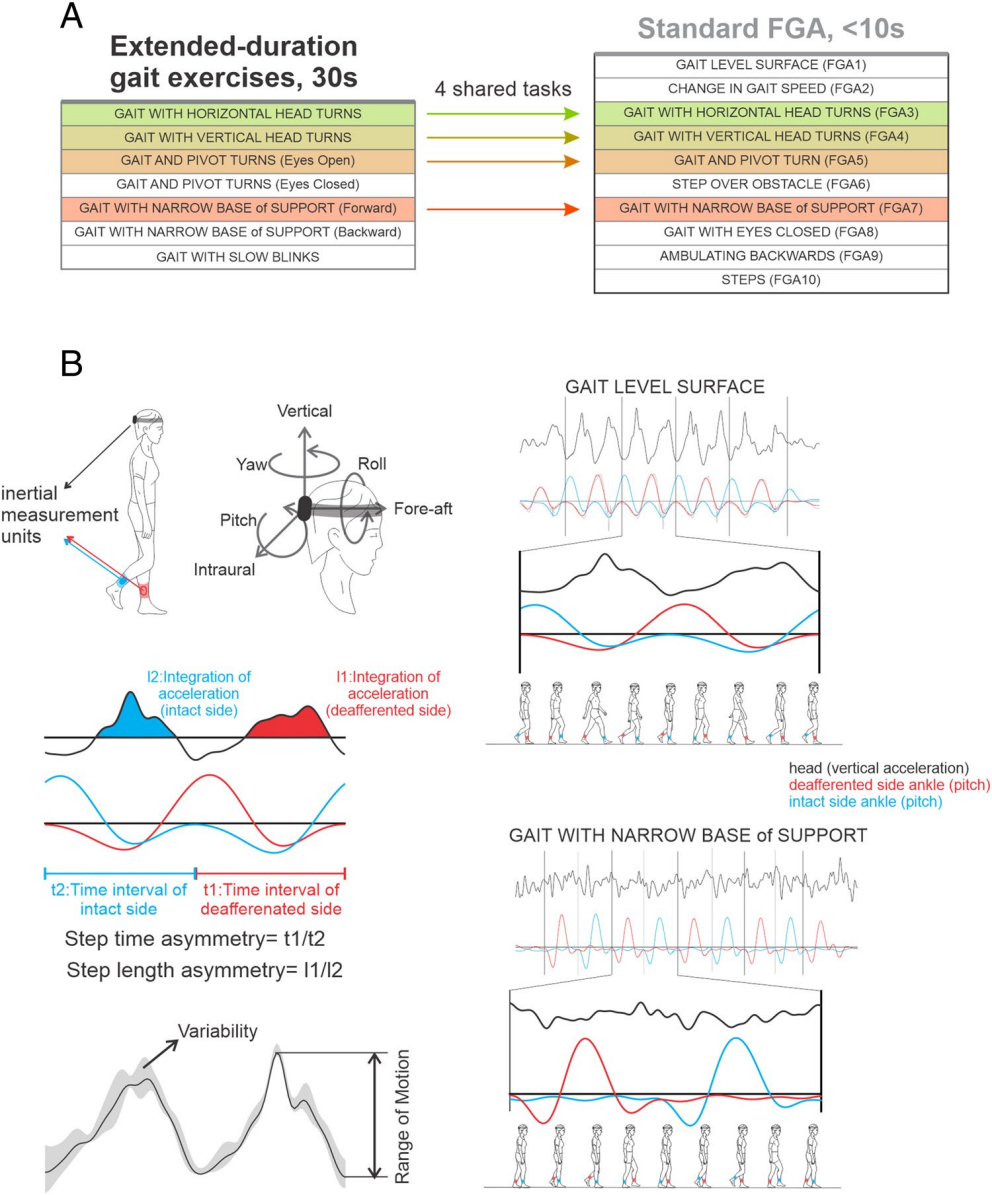
Summary of main clinical and kinematic outcome measurements. (**A**) 4 of the 10 standard Functional Gait Assessment (FGA) tasks, as well as 3 traditional vestibular gait exercises were completed for an extended 30-s time duration in addition to the 10 standard FGA tasks, each completed for ~ 10 s, a relatively short duration. (**B**) Kinematic measures were captured with Shimmer IMUs among 6 dimensions of head motion (translational acceleration of the fore-aft, lateral, and vertical axes and angular velocity along the yaw, pitch, roll planes) with step cycle segmentation using the ankle sensor. Additionally, step cycle asymmetry as well as variability of range of motion was captured among the intact vs deafferented side.

The FGA is a measure of dynamic postural stability and includes 10 walking tasks^30,31^: (1) Gait on a level surface, (2) Change in gait speed, (3) Gait with horizontal head turns, (4) Gait with vertical head turns, (5) Gait with a single 180° pivot turn, (6) Gait while stepping over an obstacle, (7) Gait with narrow base of support (i.e., 10 consecutive heel to toe steps), (8) Gait with eyes closed, (9) Backwards gait, and (10) Steps. Figure 1A. An experienced clinician subjectively rated the participants ability to perform each task, with scores ranging from 0 to 3 points. All participants performed the FGA independently, for a single trial, without assistance of a device or of the clinician. The participant’s gait speed was recorded for each task (i.e., distance achieved/time (s) required to complete each task).

### Physiological measures

#### Video head impulse test (VHIT)

Vestibular ocular reflex (VOR) gain (eye velocity/head velocity) was measured using the ICS Otometrics system (Natus Medical Incorporated, Denmark). Participants were seated 1 m from a stationary visual target, in room light. Right eye velocity and head velocity were sampled at 220 Hz during 12 passive head rotations among both directions of each of the three semi-circular canal planes: yaw, left anterior/right posterior (LARP), right anterior/left posterior (RALP). vHIT traces were deleted if the eye velocity trace preceded head velocity, or if the passive head rotation trace did not match the acceleration profile suggested by the manufacturer. VOR mean gain values were calculated based on the area under the curve of eye/head velocity. VOR gains within 0.8–1.2 with standard deviation < 0.12 were considered normal^32,33^. We did identify corrective saccade metrics (i.e., latency, amplitude of the covert and overt saccades as well as PR score). Yet, given we found no significant correlations in our analyses, we opted not to include the corrective saccade metric data in our methods and results.

### Subjective measures

Participants rated self-perceived severity of symptoms of dizziness, imbalance, headache, and anxiety using the Dizziness Handicap Inventory (DHI)^34,35^, Activities Balance Confidence Scale (ABC)^35–37^, as well the Headache Impact Test (HIT)^38^, and Beck Anxiety Inventory (BAI)^39^.

### Vestibular rehabilitation gait exercises

In addition, to the short-duration FGA exercises detailed above, we also asked participants to perform 7 traditional vestibular rehabilitation gait exercises, for a single trial, to assess how these tasks can distinguish between VS patients and healthy controls (Fig. 1A). These tasks included 7 exercises for a 30-s duration each. Among the 7 extended duration exercises, 4 tasks were both performed for ~ 10 s during the FGA and then repeated for a 30 s duration. These 4 exercises included: (1) gait with horizontal head turns, (2) gait with vertical head turns, (3) gait with pivot turns, and (4) gait with narrow base of support forward (i.e., tandem walking). The remaining 3 exercises that were not included in the FGA included: (1) gait with pivot turn, eyes closed, (2) gait with narrow base of support backward (i.e., tandem backward walking), (3) gait with slow blinks (1 step with eyes open, 2 steps eyes closed).

### Kinematic measures

Participants completed the seven 30-s vestibular rehabilitation gait exercises as well as the 10 short-duration FGA tasks while wearing 6 (51 mm × 34 mm × 14 mm) micro-electromechanical (MEMS) sensors (Shimmer3 inertial measurement unit, Shimmer Research, Dublin, Ireland) (Fig. 1B). Three of the sensors used for analysis were securely and comfortably attached to the participants’ head (with the IMU inserted securely inside a tight pocket of an elasticized head band and positioned at posterior aspect of the head), left ankle, and right ankle. The other three sensors, as mentioned above, were attached to the upper and lower trunk and dominant hand. The trunk and limb sensors were attached within a plastic clip within an elastic band and secured with additional tape. Quantitative data were sampled at 500 Hz and recorded on a built-in micro SD card. The pitch rotational velocity of the ankle sensors was used to detect ipsilesional and contralesional step cycles (Fig. 1B). The step cycle duration and head kinematic measures were then computed across ipsilesional step cycles. The total head range of motion, standard deviation, as well as coefficient of variation (CV), along each of 6 dimensions of head motion (translational acceleration of the fore-aft, lateral, and vertical axes and angular velocity along the roll, pitch, and yaw) were computed. Step length asymmetry were defined as the ratio between the integration of head vertical acceleration measured from the ipsilesional over the contralesional step cycles. Step time asymmetry was defined as the ratio of the time interval between ipsilesional over contralesional step cycles of the right over left step cycles in healthy controls (Fig. 1B**)**.

We also computed global kinematic scores based on the three computed kinematic measures (total head range of motion, standard deviation, and CV, see above) in 6 dimensions from the 4 shared tasks that were performed both as part of the extended duration gait exercises (30 s) and the standard FGA tasks (< 10 s). The global kinematic score ranged between 0 (most severely impaired) to 100 (normal) and was computed as follows: (i) each selected kinematic measure (e.g., range of motion for vertical acceleration) during each task was normalized using a linear transformation of mean ± 2SD to a number between 0 and 100 (i.e., normalized mean = 50 and normalized SD = 25); (ii) in order to keep the range of scores between 0 and 100, the outliers (i.e., numbers outside mean ± 2SD) were projected to the closest number within this range (i.e., either 0 or 100); and (iii) the normalized numbers across all three kinematic measures, 6 dimensions, and 4 tasks were then averaged. We compared head kinematic measures at the preoperative and 6-week postoperative time points and then contrasted these with head kinematic measures obtained from age-matched healthy control participants.

### Statistics

For the comparison between the kinematic measures of the vestibular participants (pre- and postoperative) and age-matched healthy controls we used a non-parametric paired sample permutation (re-randomization) test. Specifically, we generated 2000 randomized rearrangements of the observed data points and then computed p-values of the actual observed measures. Pearson correlations were used to compute the correlation coefficients and p-values of the correlations between kinematic and clinical measures. To find the consistent trends across several exercises we assessed whether the correlation for the majority of exercises (i) were significant (p < 0.05) and (ii) had the same sign (i.e., correlation was consistently positive/negative across exercises). Correction for multiple comparisons were not performed since the goal of this exploratory study was to investigate unilateral vestibular participants already known to be different from healthy controls based on clinical assessment; performing correction would have exaggerated Type II errors. Throughout the text, values are expressed as mean ± 1 SD and significance is reported at p < 0.05. All data processing and statistical tests were performed using MATLAB (The MathWorks, Inc., Natick, Massachusetts, United States).

## Results

### Kinematic and temporal measures during gait distinguish vestibular schwannoma participants from healthy controls

**Figure 2:**
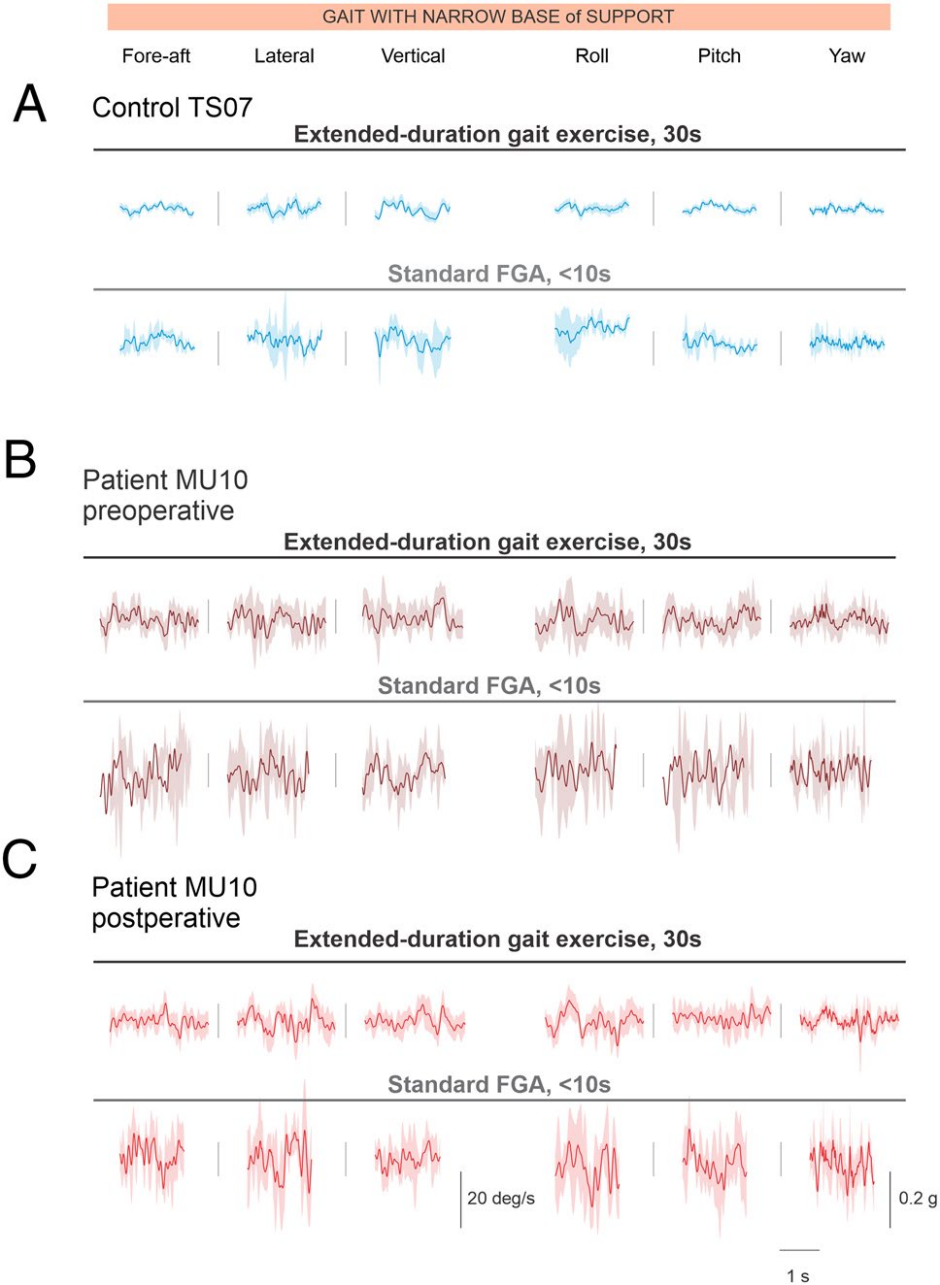
Example of head movement in 6 axes of motion. (**A**) Head movement recorded from a typical healthy control during a 30-s extended (top) as well as a short-duration task (bottom) of gait with a narrow based of support, which is a standard task within the FGA. (**B**,**C**) similar to (**A**) for a typical VS patient, pre- and postoperatively, respectively.

Figure 2 illustrates head motion across gait cycles, in all 6 translational (i.e., Fore-aft, Lateral, and Vertical) and rotational (i.e., Roll, Pitch, and Yaw) axes, for a typical healthy control subject (Fig. 2A) and for an example participant with VS preoperatively and postoperatively (Fig. 2B,C). The VS patient demonstrated in Fig. 2B had post-operative VOR mean gains that were significantly reduced among all 3 ipsi-lesional semi-circular canal planes with the presence of overt corrective saccades relative to the pre-operative time point. For this reason, we felt this participant was representative of the head kinematics expected pre and post operatively of a VS patient and the VS group. Data are shown for the task “gait with a narrow base of support” from both the extended-duration 30-s gait exercise (top panels) and standard FGA (bottom panels). As shown in this figure, the example VS participant—both pre and postoperatively—experienced larger and more variable head movements compared to the healthy control.

**Figure 3:**
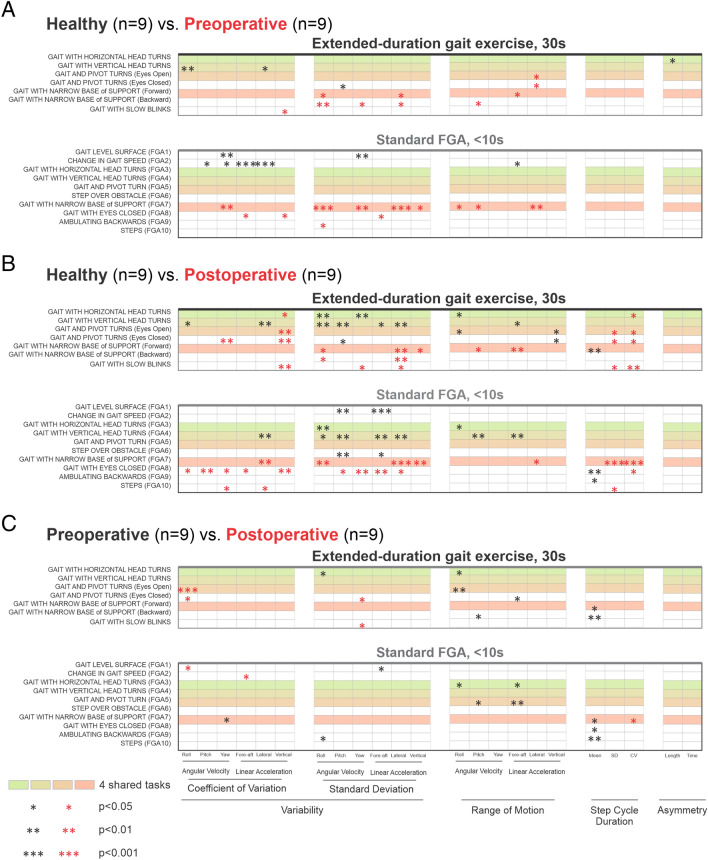
Comparison of kinematic measurements between (**A**) healthy controls and pre-operative participants, (**B**) healthy controls and post-operative participants, (**C**) preoperative and postoperative VS participants during the 7 traditional extended duration (30 s) gait exercises (top) and the 10 standard Functional Gait Assessment (FGA) short duration (< 10 s) tasks (bottom). 4 of 7 gait exercises were an extended-duration of similar FGA tasks (colored rectangles). Asterisks indicate difference at 3 significance levels (*0.05, **0.01, ***0.001). (**A**,**B**) Black asterisks indicate the healthy controls had a larger value than the VS group. Red asterisks indicate the VS participants had a larger value than the healthy control participants. (**C**) Black asterisks indicate the preoperative VS participants had a larger value than the postoperative VS participants.

The kinematic data illustrated in Fig. 2 provide a snapshot, for an individual participant, of the results shown in Fig. 3**,** which summarizes the kinematic measures computed for each of the 7 gait exercises (top panels) and 10 standard FGA tasks (bottom panels) for all participants. These measures include the variability [coefficient of variation (CV) and standard deviation (SD)] and range of head motion for all 6 axes, average and variability of the step cycle duration, and time and length asymmetry (see “Methods”). The four 30-s vestibular gait exercises that were extended-duration versions of standard FGA tasks are highlighted in color. We found that the range of motion and variability of head motion were both altered for the extended duration gait exercises and standard FGA tasks in both pre- and post-operative VS participants as compared to healthy controls (Fig. 3A,B, black and red asterisks). However, variability measures were generally more informative for the standard FGA as compared to the gait exercises, particularly for postoperative VS participants (Fig. 3B, two left columns). In contrast, range of motion measures were more informative for the extended gait exercises as compared to the standard FGA tasks, and again this was particularly the case for postoperative VS participants (Fig. 3B, middle column). In contrast, we found only small differences between the kinematic measures of pre- and postoperative VS participants for both standard FGA and gait exercises (Fig. 3C).

Finally, in addition to the observed differences in kinematic measures described above, we further found that step cycle duration and its variability distinguished VS participants and healthy controls (Fig. 3). Specifically, step cycle duration was significantly longer (i.e., slower gait) in postoperative than in preoperative VS participants and healthy controls during several of the more challenging tasks (i.e., “gait with narrow base of support,” “gait with eyes closed,” and “ambulating backwards”) across conditions. The step cycle duration of postoperative VS participants was also more variable than healthy controls. We did not, however, find any differences between the step cycle durations of preoperative VS participants and healthy controls. Moreover, our asymmetry analysis did not reveal differences between three groups in either time or length asymmetry (Fig. 3, right column).

### Computing a global kinematics score to distinguish vestibular schwannoma patients from healthy control participants

**Figure 4:**
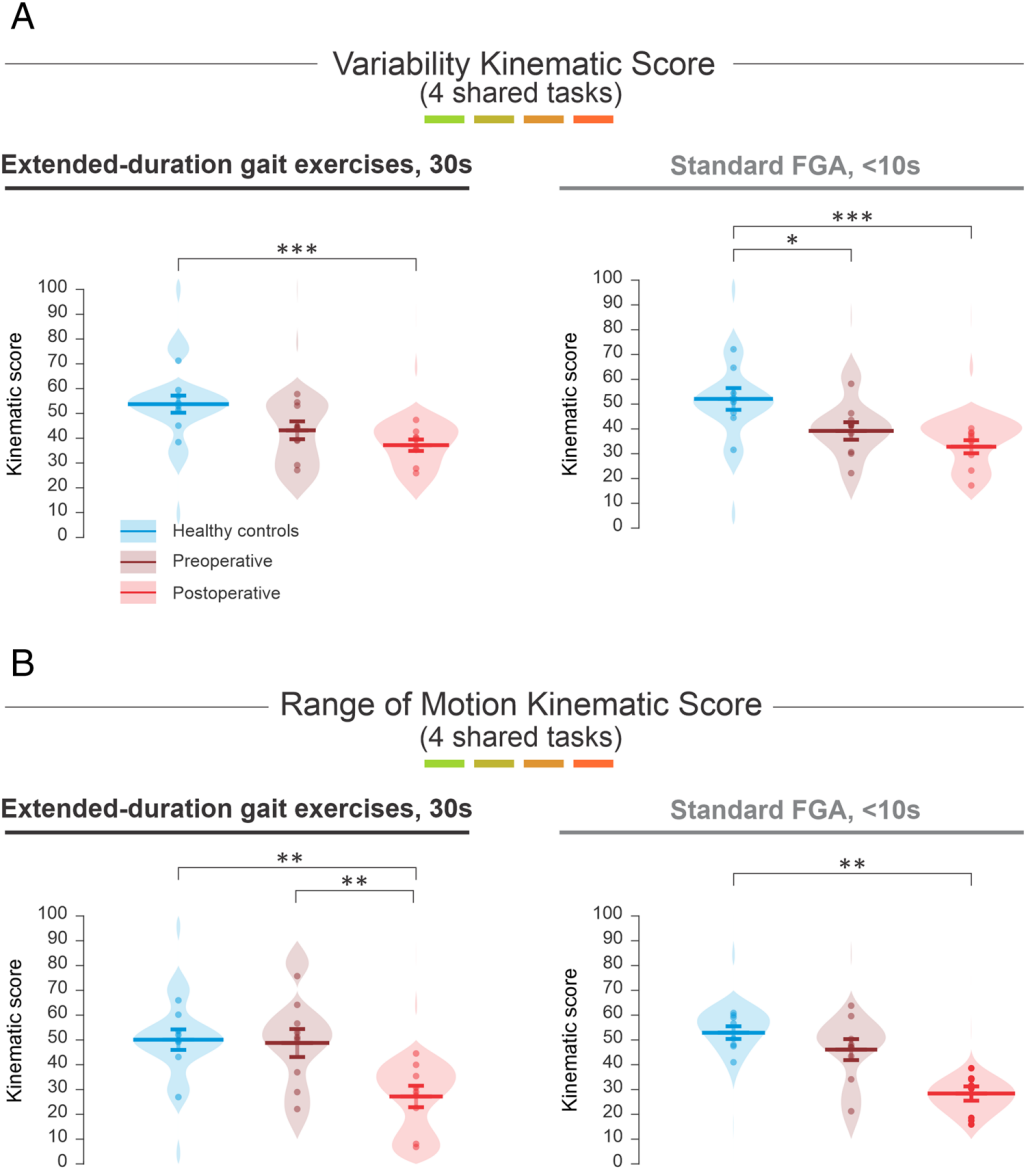
A computation of a global kinematic score of the FGA which significantly distinguished post-operative VS participants from healthy controls. (**A**) the kinematic scores computed from the variability measures during extended duration gait exercises (30 s) as well as all FGA tasks (< 10 s) for the healthy controls (blue), preoperative VS participants (dark red), and postoperative VS participants (red). (**B**) the kinematic scores computed from the range of motion measures during extended duration gait exercises (30 s) as well as all FGA tasks (< 10 s) for the healthy controls (blue), preoperative VS participants (dark red), and postoperative VS participants (red). Vertical lines correspond to the mean ± SEM (Standard Error of the Mean) of the kinematic score for each group. The width of the shaded areas corresponds the probability of the kinematic score across each group. Asterisks reflect significant difference between healthy control participants and pre and postoperative participants (*p < 0.05).

Figure 4 illustrates global kinematic scores that were computed from variability (Fig. 4A) versus range of motion (Fig. 4B) measures (see Methods). First, as shown in Fig. 4A, the kinematic score computed based on variability measures distinguished postoperative VS participants from healthy control participants (Fig. 4A, left panel, p = 0.001). However, there was no significant difference in this score for healthy controls versus preoperative VS participants (Fig. 4A, left panel, p > 0.05). In contrast, for the shorter duration standard FGA tasks, this variability-based kinematic score was significantly different for both pre and postoperative VS participants relative to healthy controls (Fig. 4A, right panel, p = 0.037 and 0.001, respectively). Thus, the variability kinematic score during short duration, standard FGA tasks was more informative in distinguishing preoperative VS participants from healthy controls.

In contrast, the kinematic score computed based on range of motion measures was informative in distinguishing preoperative from postoperative VS participants for the four extended duration, 30-s, gait exercises (Fig. 4B, left panel, p = 0.009). In contrast, this score was not different between the preoperative VS participants and healthy controls for the four corresponding short-duration standard FGA tasks (Fig. 4B, right panel, p > 0.05). Additionally, similar to the variability kinematic score (i.e., Fig. 4A), the range of motion kinematic score was significantly different for VS postoperative versus control participants during both gait conditions (Fig. 4B, left and right panels; p = 0.002 versus p = 0.005, respectively for extended versus FGA gait). Thus, in summary, the variability kinematic score, was more informative for the short duration, standard FGA (< 10 s), while the range of motion kinematic score, was more informative for the extended-duration gait exercises (30 s).

### Preoperative kinematic measures were correlated with functional clinical measures

**Figure 5:**
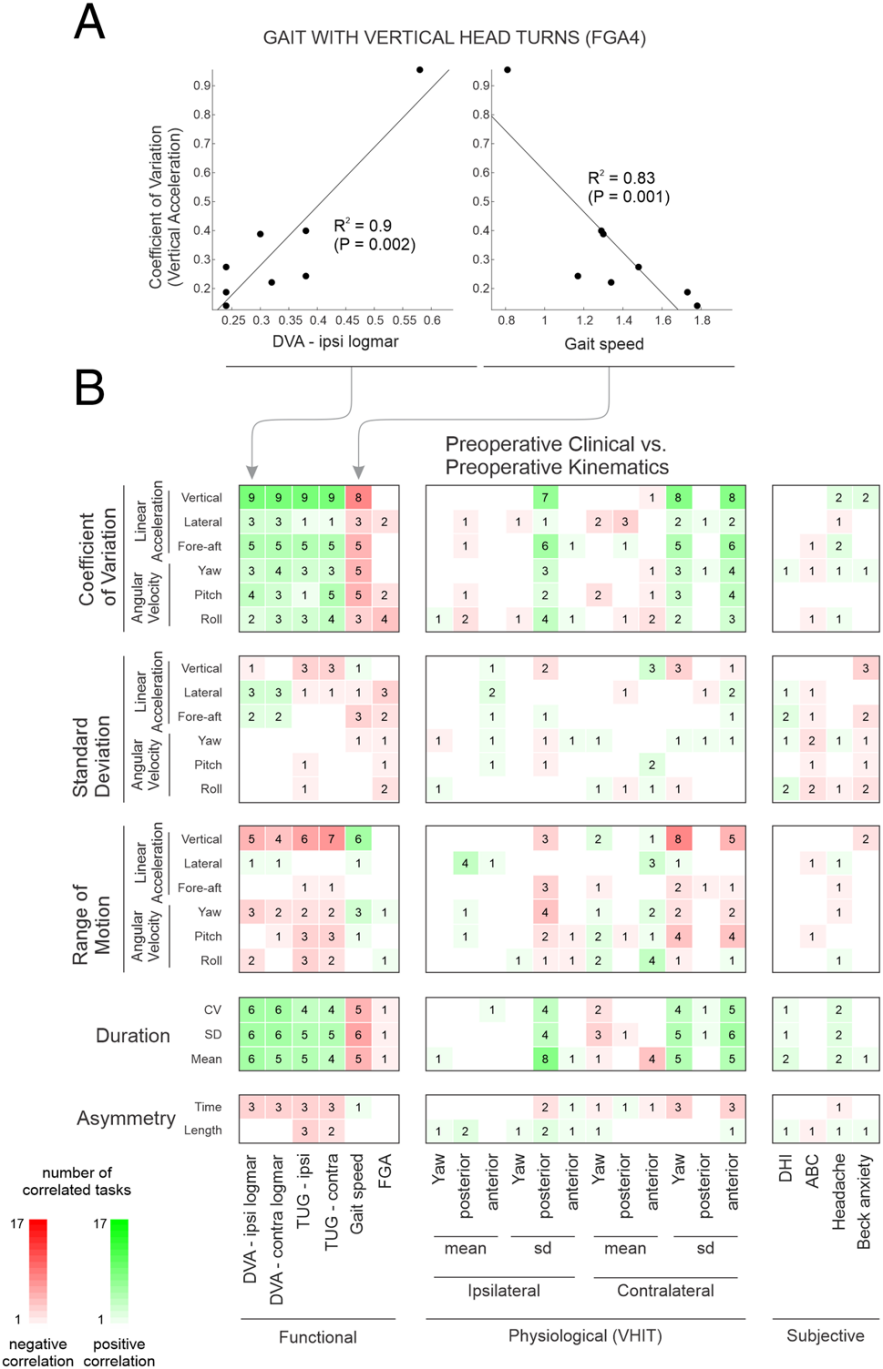
The correlation between the clinical and kinematic measure preoperatively. (**A**) Scatter plots reflecting the correlations between the pre-operative head kinematic vertical acceleration coefficient of variation and DVA—ipsilesional logMAR (left panel) and gait speed (right panel). (**B**) Correlation of pre-operative clinical vs head kinematic measures among 6 dimensions. Green and red squares reflect positive and negative correlations, respectively. Brightness and number of squares indicate the number of exercises (1–17) with a significant correlation (p < 0.05).

We next asked whether the objective head movement kinematic measures described above for VS participants could predict clinical measures (functional, physiological, and subjective). To address this question, we evaluated the correlations between clinical measures and head kinematic measures computed for the 7 extended gait exercises as well as 10 standard FGA tasks (see “Methods”). Supplementary Tables 2 and 3 summarize the VS pre and post operative clinical outcomes data. The plots in Fig. 5A illustrates example correlations observed for preoperative VS participants. Specifically, in VS preoperative participants, the variability of vertical head motion (CV) in the “gait with vertical head turns” during extended gait exercise demonstrated positive and negative correlations with DVA logMAR scores and gait speed, respectively (Fig. 5A). Figure 5B, Fig. 5, Supplementary 1 summarize the significant relationships observed between kinematic measures (vertical axis) and the clinical measures (horizontal axis), where the numbers shown within each of the colored squares indicates the number of exercises for which there was significant correlation. Overall, we found that certain clinical measures, notably DVA, TUG, gait speed, were the most consistently correlated with the head kinematic measures. We also found some correlations between the physiological (e.g., variability of the VOR gain) and kinematic measures (e.g., step cycle duration, range and variability of vertical head motion). However, for the majority of tasks we did not find correlation between subjective and kinematic measures (Fig. 5B). Comparable results were seen when we analyzed the standard FGA (< 10 s) tasks and extended-duration (30 s) gait exercises separately (Fig. 5, Supplementary 1, 2).

### Postoperative kinematic measures were correlated with functional clinical measures

**Figure 6:**
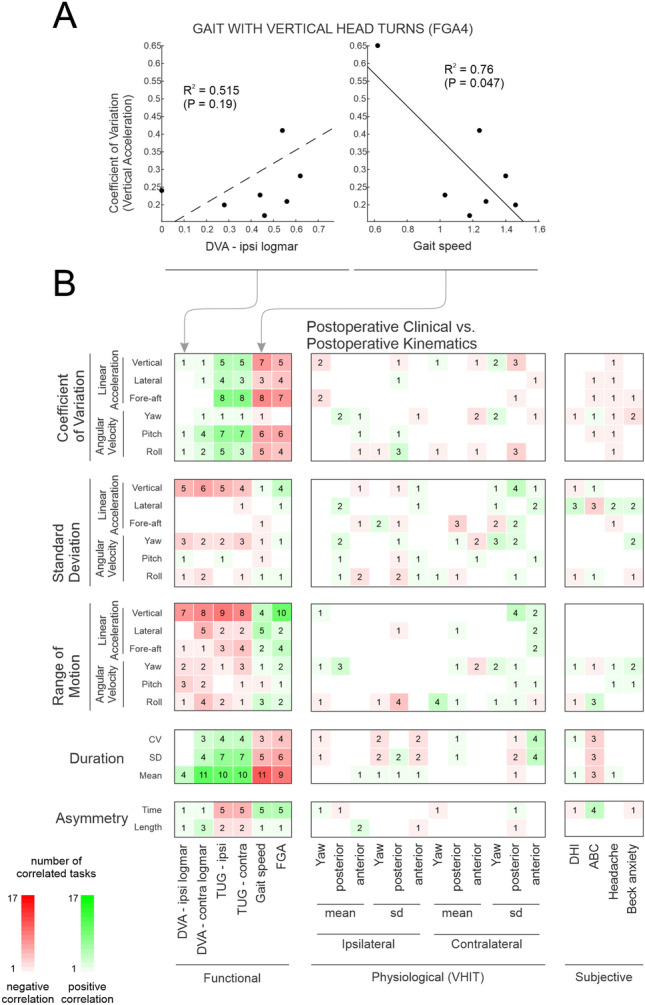
The correlation between the clinical and kinematic measure postoperatively. (**A**) Scatter plots reflecting the correlations between the postoperative head kinematic vertical acceleration coefficient of variation and DVA—ipsi logMAR (left panel) and gait speed (right panel). (**B**) Correlation of post-operative clinical vs head kinematic measures among 6 dimensions. Green and red squares reflect positive and negative correlations, respectively. Brightness and number of squares indicate the number of exercises (1–17) with a significant correlation (p < 0.05).

Finally, we completed a complementary analysis of the relationships between head kinematic measures during the 7 gait exercises and 10 FGA tasks, and clinical measures after surgery (Fig. 6). The plots in Fig. 6A illustrate the same examples of relationships as shown above for these same participants before surgery (Fig. 5A). The variability of vertical head motion (CV) for “gait with vertical head turns” during extended gait exercise demonstrated negative correlations with gait speed after surgery (Fig. 6A, right panel), whereas there was no significant relationship with DVA logMAR scores (Fig. 6A, left panel). Figure 6B and Fig. 6 Supplemental 1 summarize the significant relationships observed between kinematic measures (vertical axis) and the clinical measures (horizontal axis), for VS participants after surgery. Comparable results were seen when we analyzed the extended-duration gait exercises and shorter-duration, standard FGA (< 10 s) tasks separately (Fig. 6 Supplemental 1, 2). Overall, as observed above for preoperative VS participants, kinematic measures of VS postoperative participants were most predictive of postoperative functional measures including, TUG, gait speed, and FGA scores. Variability of vertical head motion showed the highest numbers of correlations. Interestingly, postoperative VS participants with worse functional clinical measures generated longer step cycle durations as well as smaller, more variable vertical head motion. Moreover, while VS postoperative kinematic measures were predictive of postoperative functional clinical measures, they were less predictive of postoperative physiological and subjective measures.

## Discussion

To our knowledge, this is the first study to have compared head kinematics using IMUs in six dimensions during gait tasks performed over an extended, 30-s period (gait rehabilitation exercises), versus gait tasks performed for a short, 10-s duration (via the standard FGA). Additionally, this study is the first to have utilized kinematic measures recorded during either extended duration exercises or short duration gait tasks to predict clinical measures in individuals with vestibular loss (pre and post vestibular deafferentation).

We found that: (i) head motion variability and range of motion is altered in VS participants relative to controls during several extended duration gait exercises and short duration FGA tasks, both pre and postoperatively. (ii) Only minor differences were observed when the same comparisons were made between pre and postoperative groups. (iii) Head kinematics and step cycle duration were predictive of clinical measures (specifically, DVA, TUG, FGA, gait velocity) for both gait task groups. (iv) A global kinematic score computed from variability measures, among the 4 shared short duration FGA gait tasks was more informative than that computed for the corresponding 4 extended duration gait exercises. (v) In contrast, a global kinematic score computed from range of motion measures was more informative in distinguishing VS (both pre and postoperative) from healthy controls when computed across the 4 shared extended gait exercises as compared to the corresponding shorter FGA tasks. This change in range of motion is suggestive of increased dynamic instability^40,41^ and was not paired with significant lengthening of gait cycle duration, which otherwise would have been considered a more conservative, protective gait strategy (e.g., increased range and duration). We speculate that this behavior likely puts VS participants at a greater risk of falls during extended gait tasks, where balance control could be compromised by greater gait cycle instability^41^ uncoupled from compensatory temporal modifications in cycle duration, leading to slower gait speed.

In the context of VS, the slow growth of the tumor allows for the putative development of motor strategies that would enable patients to remain functional through most activities of daily living. Individuals with vestibular loss rely on adaptive or compensatory mechanisms including visual and somatosensory substitution as well as changes in feedforward motor control^42–44^. The dynamic upweighting of these extra-vestibular sensory (e.g., visual and somatosensory) signals, as well as inputs from motor pathways is evident even at the first central stage of sensory motor processing^45–48^. The strategy of implementing such available adaptive central mechanisms likely evolves over the course of tumor growth in response to the progressive impairment. Thus, an individual’s adaptative strategy may not be optimal when considered over longer durations and/or for compensating for additional perturbations such as vestibular deafferentation. Indeed, recent reports describing head movement kinematic in pre versus postoperative VS patients during gaze and balance exercises are consistent with this view^23,49^.

We speculate that relative increase in variability of VS participants for short versus extended duration gait tasks reflects that their postural maintenance follows a kinematic sequence that is initially characterized by greater movement to movement variability (i.e., motor exploration)^50^, followed by a second adaptive phase characterized by a task-dependent kinematic strategy wherein range of motion increase with the extent of challenge imposed by the task (e.g., longer duration, etc.). Our present results, focused on extended duration exercises, suggest that while gait cycle variability was present in this latter phase, the outputted gait cycle amplitude was also significantly enhanced in what could be considered a second, additional compensation strategy. Our results thus suggest that maintaining stability during extended gait recruits this latter feature of an evolving kinematic strategy, which like the initial phase of gait, is more challenging for VS participants. Interestingly, our present results align with previous reports of dynamic regulation of sensorimotor integration in human postural control, in which the repetition of a motor task (here the extended gait exercises) is taken as means towards reducing movement-to- movement motor variability, yet can potentially decrease final performance when there is inadequate regulation of corrective responses^51^. This abnormal motor response was likely aggravated in our study population by the biased influence of the tumor over what had been, prior to the presence of the VS tumor, a normal state of the neural circuitry that together with the locomotor apparatus modulate the feed-forward information process warranted in effective, healthy locomotor adaptation^52^. One could speculate that the kinematic changes observed overtime were unrelated to changes in motor control adaptative strategies, but rather were the result of biomechanical entrainment to the task.

In the present study we chose to focus on head kinematics because head movements not only activate the vestibular sensors, but they are also modulated by vestibular-motor pathways. Furthermore, it has been proposed that humans strongly rely on head-based orientation for inertial guidance^13,53,54^. However, few studies have focused on head kinematics during gait in VS participants. Paul et al. studied 1D head kinematics (yaw axis) and reported reduced purposeful movement in postoperative VS participants during gait including the FGA^22^ and community ambulation^19^, compared to controls. To date, only a single study^24^ has quantified 6D head kinematics in VS participants during the standard FGA. Importantly, several of the main findings of our present study are consistent with those of this prior study, which focused on a separate population of VS participants. Notably, here as well as this prior report, VS participant head kinematics differed from those of controls, both before and after surgery. As reviewed above, here we have further established that while measures of variability are more informative among the short duration FGA tasks, measures of range of motion are more informative for extended duration exercises.

Finally, here we have further shown that head kinematics and step cycle duration—both during short duration FGA and extended duration gait rehabilitation exercises—are predictive of several clinical measures, but not of subjective quality of life assessments. In this context, our findings add to a growing body of work demonstrating comparable conclusions based on head movements during other classes of vestibular exercises (i.e., gaze^55,56^ and balance^49^). Moreover, we found that kinematic measures from both short and extended duration conditions better predicted physiological (i.e., vHIT) measures preoperatively than postoperatively. Thus, although the duration of the presence of the vestibular schwannoma tumor and the VS participants’ level of central adaptation, prior to participation, was unknown, our exploratory findings suggest that the quantification of 6D kinematics across multiple time points can be utilized to predict clinical outcomes in VS pre and/or postoperative state, as well as for informed exercise prescription in the future.

## Conclusion

Taken together, our findings reveal vestibular schwannoma patients alter their head variability and range of motion during short and extended duration gait tasks pre and postoperatively in contrast to healthy controls. Variability and range of motion kinematic measures provide different information regarding the strategy individuals deploy to maintain functional locomotion. We also found that kinematic measures are predictive of functional gait clinical measures in individuals with vestibular deafferentation. The global head kinematic score may prove useful among future studies in individuals with vestibular loss across multiple time points and levels of rehabilitative progression.

### Supplementary Information

Supplementary Information.

## Data Availability

The datasets used and/or analysed during the current study available from the corresponding author on reasonable request.
