# Supplementary material for: Head movement kinematics are differentially altered for extended versus short duration gait exercises in individuals with vestibular loss
Source: Sci Rep. 2023 Sep 27;13:16213. doi: 10.1038/s41598-023-42441-2 (PMC10533850; doi:10.1038/s41598-023-42441-2)
Supplement: Supplementary file 1 — Supplementary Information. [file 41598_2023_42441_MOESM1_ESM.pdf]

Head movement kinematics are differentially altered for extended versus short duration gait exercises in individuals with vestibular loss

Jennifer L. Millar[1^], Omid A. Zobeiri[2,3^], Lin Wang[2], Wagner H. Souza[1,2], Michael C. Schubert[1,4], Kathleen E. Cullen[2,4,5,6,*]

Author Affiliations:

[1]Department of Physical Medicine and Rehabilitation, Johns Hopkins University School of Medicine, Baltimore, MD, USA

[2]Department of Biomedical Engineering, Johns Hopkins University, Baltimore, MD, USA

[3]Department of Biomedical Engineering, McGill University, Montreal, QC, Canada

[4]Department of Otolaryngology-Head and Neck Surgery, Johns Hopkins University School of Medicine, Baltimore, USA

[5]Department of Neuroscience, Johns Hopkins University School of Medicine, Baltimore, USA

[6]Kavli Neuroscience Discovery Institute, Johns Hopkins University, Baltimore, MD, USA

[^]These authors contributed equally: Jennifer L. Millar and Omid A. Zobeiri

***Corresponding Author**:

Kathleen E. Cullen, Johns Hopkins University School of Medicine
720 Rutland Ave, Traylor 504, Baltimore, MD 21205-2109, USA.
Email: kathleen.cullen@jhu.edu

Figure 5 - supp 1

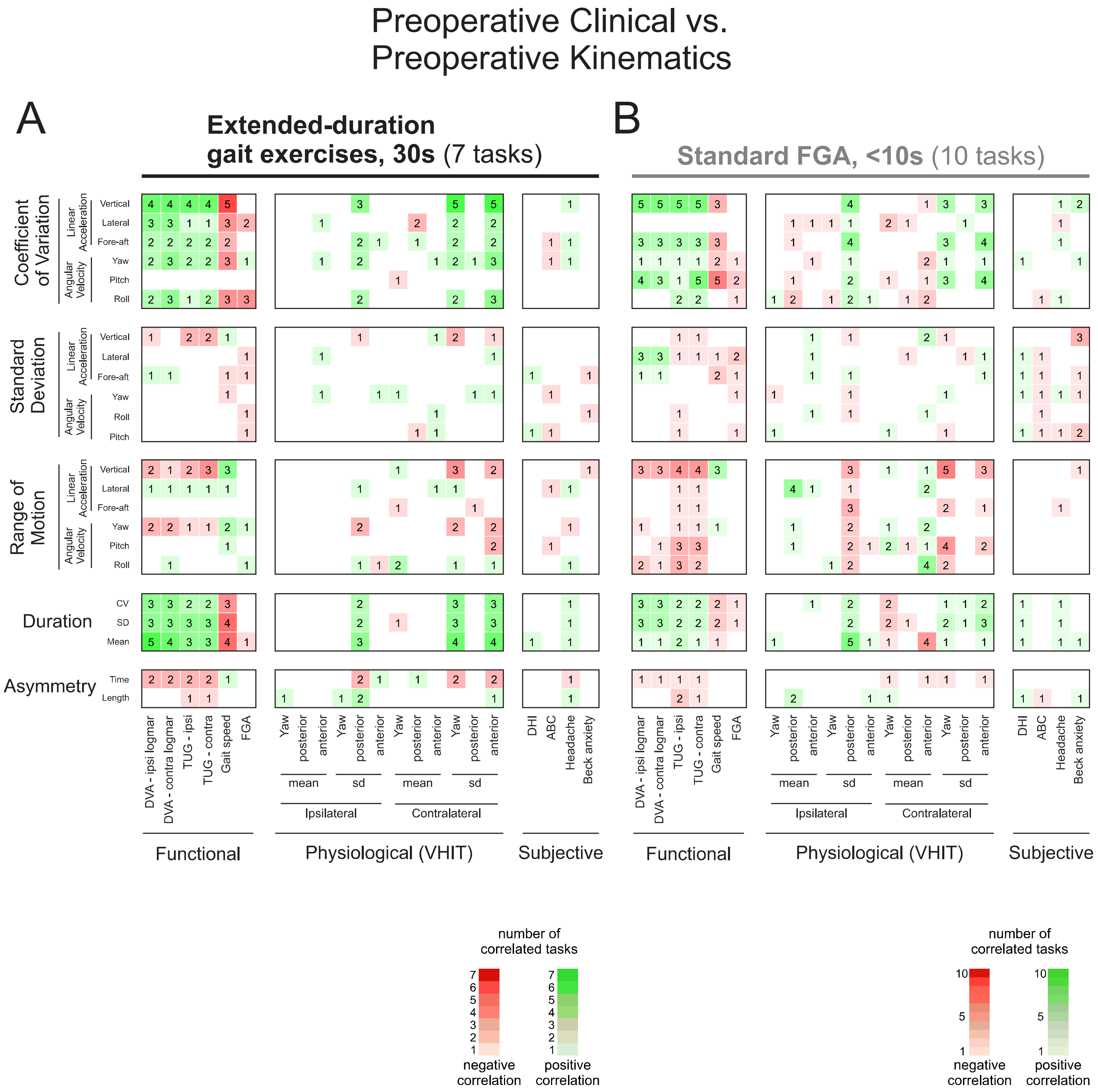

FIGURE 5 SUPPLEMENTAL 1. The correlation between the clinical and kinematic measure preoperatively for all 7 extended-duration gait exercises (A) and 10 standard FGA tasks (<10s) (B) and all among 6 dimensions. Green and red squares reflect positive and negative correlations, respectively. Brightness and number of squares indicate the number of exercises (1-10 for A, and 1-7 for B) with a significant correlation (p<0.05).

Figure 5 - supp 2

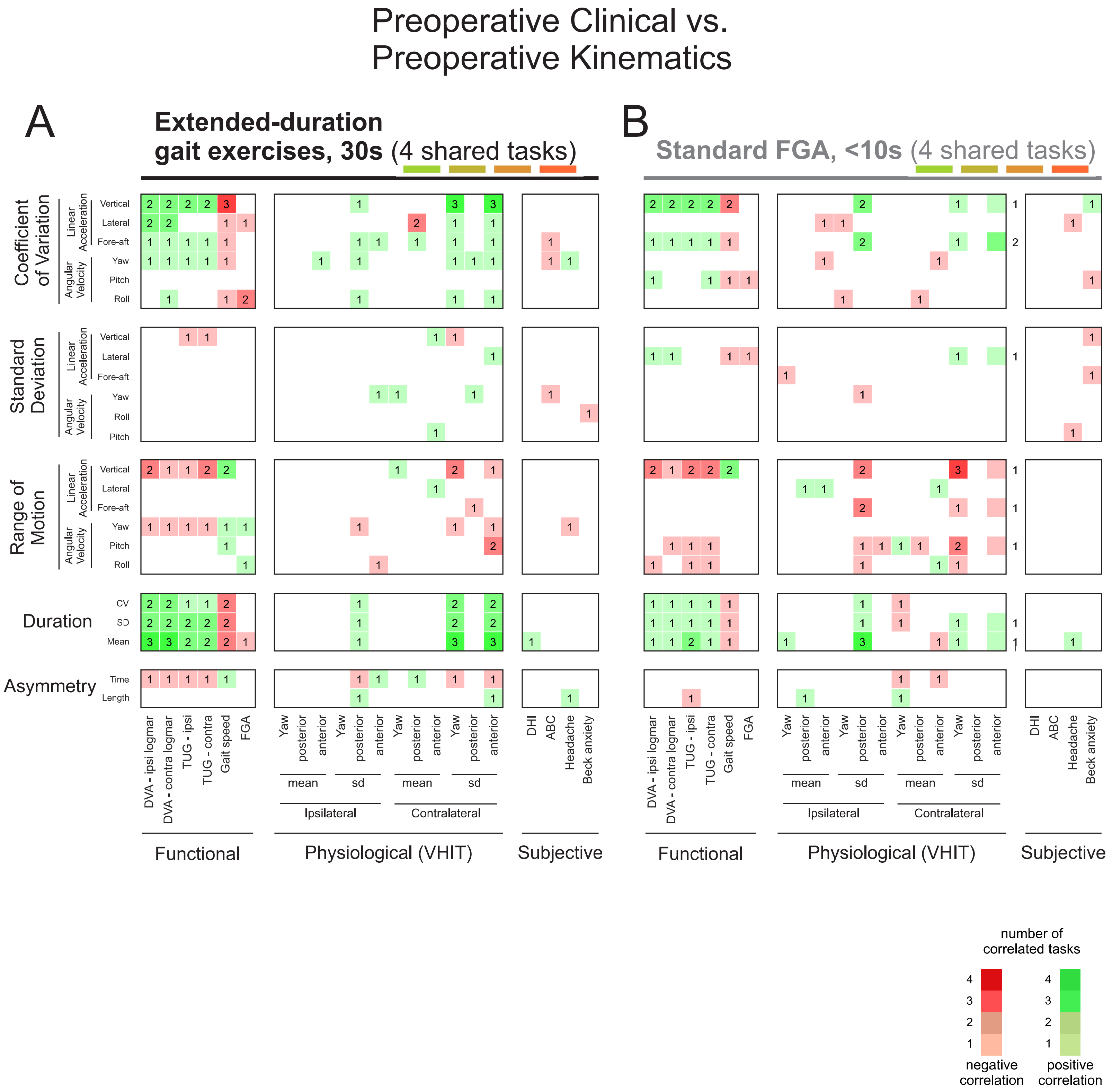

FIGURE 5 SUPPLEMENTAL 2. The correlation between the clinical and kinematic measures preoperatively for extended-duration gait exercises (30s) (A) and 4 shared standard FGA tasks (<10s) (B) and among 6 dimensions. Green and red squares reflect positive and negative correlations, respectively. Brightness and number of squares indicate the number of exercises (1-4) with a significant correlation ($p<0.05$).

Figure 6 - supp 1

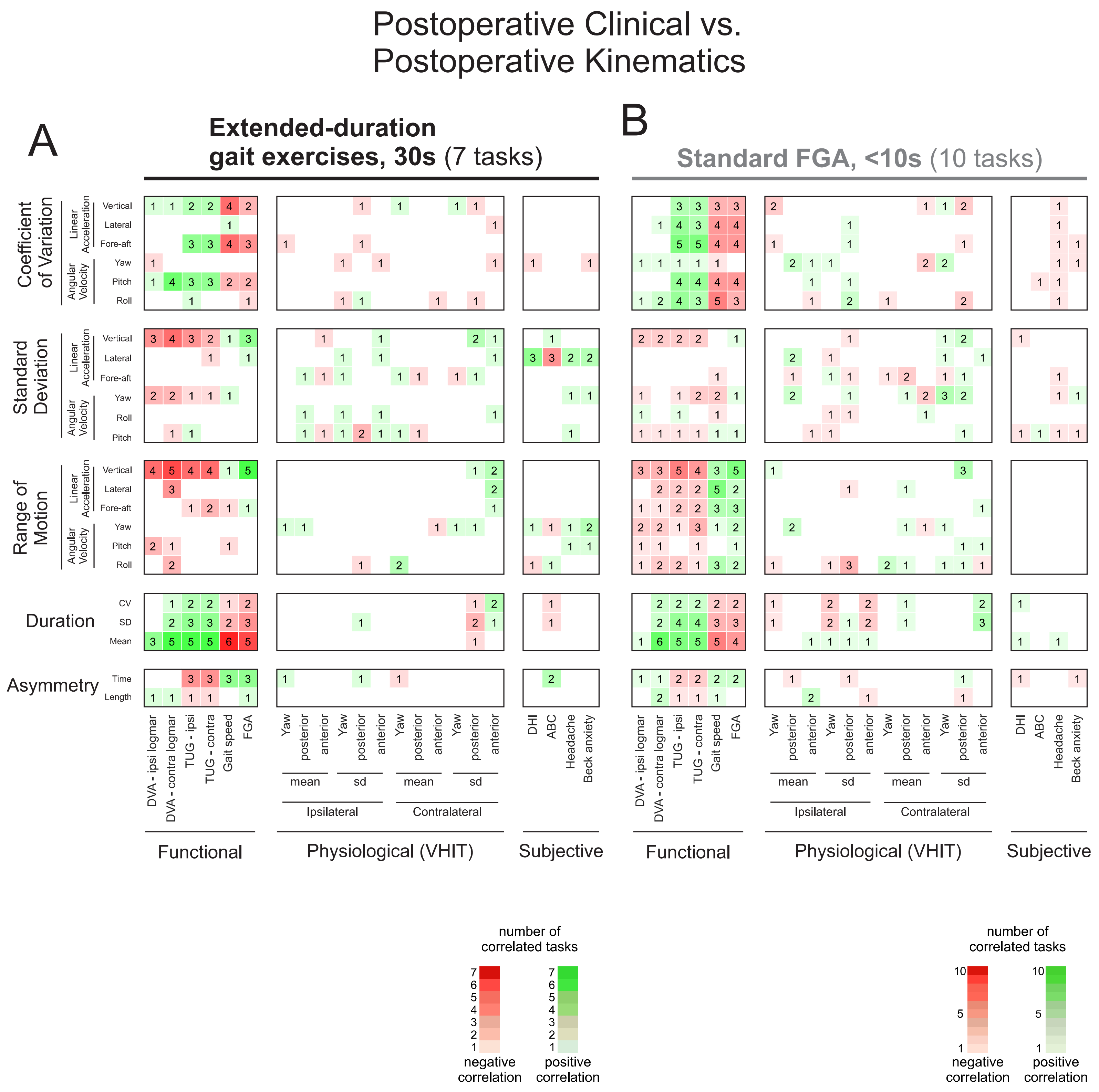

FIGURE 6 SUPPLEMENTAL 1. The correlation between the clinical and kinematic measures postoperatively for all 7 extended-duration gait exercises (30s) (A) and for all 10 standard FGA tasks (<10s) (B) among 6 dimensions. Green and red squares reflect positive and negative correlations, respectively. Brightness and number of squares indicate the number of exercises (1-10 for A, and 1-7 for B) with a significant correlation (p<0.05).

Figure 6 - supp 2

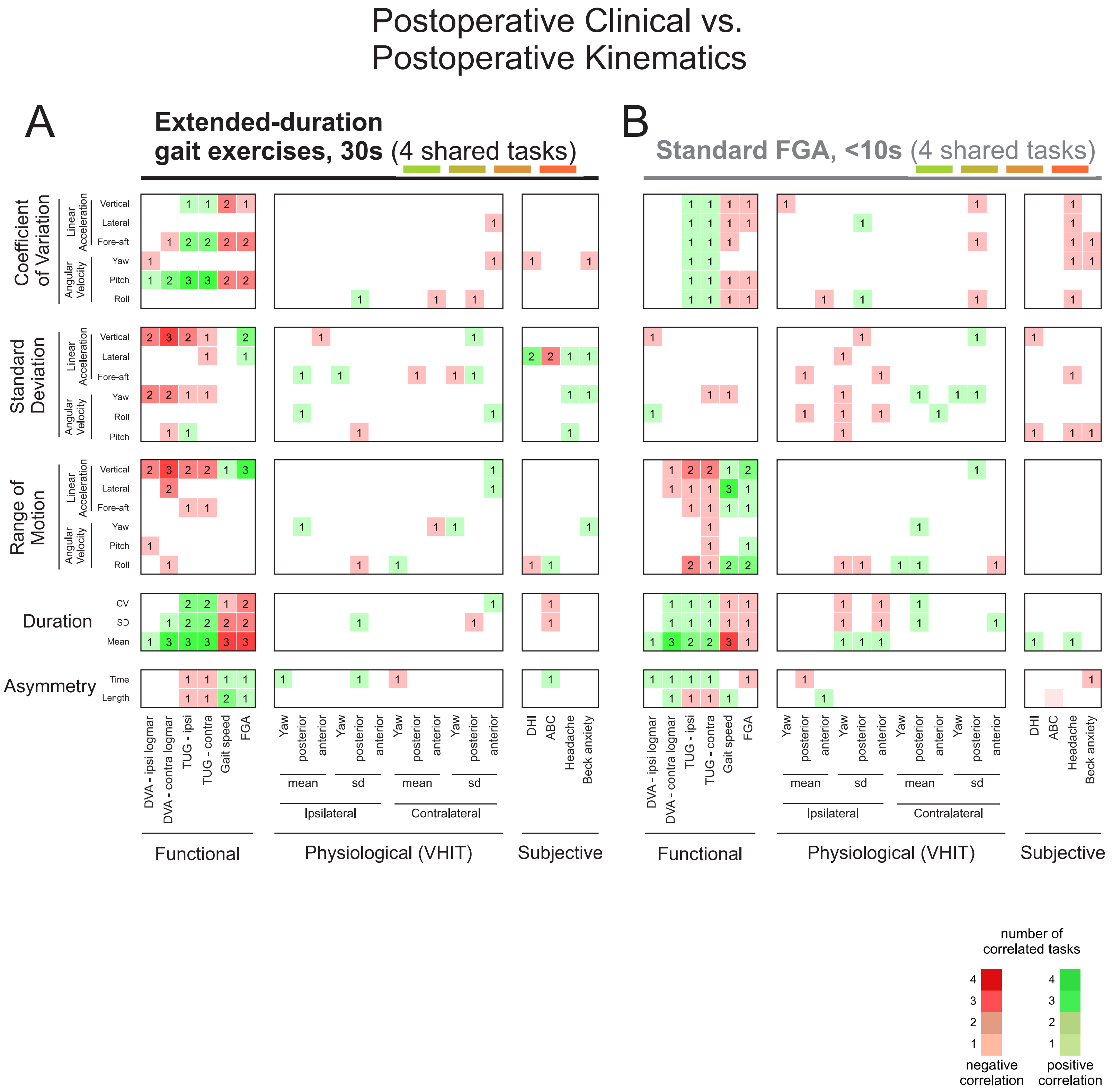

FIGURE 6 SUPPLEMENTAL 2. The correlation between the clinical and kinematic measure postoperatively for extended-duration gait exercises (A) and 4 shared standard FGA tasks (<10s) (B) among 6 dimensions. Green and red squares reflect positive and negative correlations, respectively. Brightness and number of squares indicate the number of exercises (1-4) with a significant correlation ($p<0.05$).

Table 1:

PARTICIPANT	*GENDER*	*LESION LOCATION*	*SURGERY TYPE*	*VOLUME (CM)*	*KOOS SCORE*	*AGE (YEARS)*	*HEIGHT (M)*	*WEIGHT (KG)*	*PRE-OP TESTING TIME BEFORE SURGERY (DAYS)*	*POST-OP TESTING TIME FROM SURGERY (DAYS)*
1	M	Right	SOC	1.2	3	23	1.88	131	1	40
2	M	Right	SOC	3.1	4	62	1.77	85.5	1	41
3	M	Right	SOC	1.8	2	39	1.85	102.3	11	35
4	M	Left	SOC	4.1	4	41	1.80	102.6	11	45
5	M	Right	SOC	16.9	4	49	1.82	86.1	7	45
6	M	Left	SOC	0.3	2	64	1.85	116.9	1	42
7	F	Left	SOC	1.6	3	55	1.70	81.6	17	38
8	M	Right	SOC	1.2	3	63	1.70	81.6	1	42
9	M	Left	SOC	1.8	3	70	1.78	74.8	1	44
MEAN				3.6	3.1	52±15	1.79	95.8	6	41
	M=8<br>F=1	R=5<br>L=4								

Table 1: A summary *of* VS participants' gender, VS laterality, surgery type, tumor volume, Koos score, age, height, weight, as well as number of days between testing before and after surgery. Male (M), Female (F), Suboccipital craniotomy (SOC).

Table 2:

PARTICIPANT	*VISIT TIME*	*DHI*	*ABC (%)*	*HEADACHE*	*BECK ANXIETY*	*FGA*	*TUG IPSI (S)*	*TUG CONTRA (S)*	*GAIT SPEED (M/S)*
1	PRE	0	97	48	0	29	5.7	6.3	1.49
	POST	8	100	68	0	30	5.8	5.5	1.43
2	PRE	48	57	52	19	27	7.3	6.7	1.78
	POST	38	69	56	11	28	5.9	5.5	1.46
3	PRE	6	100	46	4	30	4.1	4.3	1.63
	POST	36	81	42	3	30	5.6	5.1	1.38
4	PRE	16	96	50	12	26	5.4	5.7	1.48
	POST	44	70	57	9	23	10.2	10.2	1.40
5	PRE	40	88	64	13	21	15.1	14.6	0.81
	POST	68	23	44	7	9	24.6	22.7	0.62
6	PRE	0	100	55	2	29	7.1	7.0	1.30
	POST	10	97	45	4	29	7.7	8.1	1.24
7	PRE	0	100	44	3	30	6.3	6.4	1.41
	POST	53	60	62	11	15	7.9	NA	1.13
8	PRE	22	94	38	5	25	8.0	7.6	1.29
	POST	6	100	36	3	28	6.2	6.1	1.28
9	PRE	2	90	48	6	25	8.2	8.3	1.34
	POST	26	83	50	8	24	9.1	8.5	1.03

Table 2: A summary of VS preoperative (PRE) and 6 weeks postoperative (POST) subjective and functional measures. Activities Balance Confidence Scale (ABC), Headache Impact Test (Headache), Beck Anxiety Inventory (Beck Anxiety), Functional Gait Assessment (FGA), Timed up and Go (TUG), Ipsi-lesional (Ipsi), Contra-lesional (Contra), Gait Speed in meters/second (m/s).

Table 3:

PARTICIPANT	*VISIT TIME*	*STATIC LOGMAR*	*RIGHT DVA LOG MAR**	*LEFT DVA LOG MAR**	*VHIT LEFT YAW*	*VHIT RIGHT YAW*	*VHIT LEFT ANT*	*VHIT RIGHT POST*	*VHIT LEFT POST*	*VHIT RIGHT ANT*
1	PRE	-0.14	0.12	0.10	0.91±0.06	0.82±0.05	0.74±0.07	0.72±0.03	0.95±0.06	0.93±0.05
	POST	-0.24	0.38	0.40	0.86±0.05	0.47±0.04	0.78±0.14	0.38±0.16	1.23±0.14	0.33±0.12
2	PRE	0.00	0.22	0.24	1.07±0.07	1.16±0.06	0.49±0.15	0.52±0.21	0.89±0.14	0.37±0.06
	POST	-0.02	0.28	0.28	0.88±0.06	0.65±0.15	0.52±0.09	0.6±0.19	1.14±0.13	0.18±0.11
3	PRE	-0.30	0.68	0.68	0.86±0.05	0.79±0.02	0.52±0.14	0.64±0.03	0.82±0.07	0.85±0.05
	POST	-0.20	0.48	0.42	0.84±0.04	0.43±0.03	0.56±0.05	0.38±0.1	0.75±0.22	0.38±0.15
4	PRE	-0.20	0.28	0.24	0.61±0.09	0.8±0.08	0.29±0.12	0.75±0.07	1.15±0.01	0.95±0.14
	POST	-0.14	0.58	0.62	0.18±0.08	0.68±0.06	NA	NA	NA	NA
5	PRE	-0.08	0.84	0.58	1.0±0.05	0.46±0.32	0.66±0.11	0.51±0.11	0.5±0.33	0.34±0.25
	POST	-0.14	0.84	0.70	0.52±0.09	0.19±0.06	0.6±0.1	0.27±0.06	0.67±0.19	0.46±0.0
6	PRE	-0.20	0.32	0.30	1.02±0.07	1.01±0.05	0.97±0.09	0.86±0.2	0.76±0.19	0.92±0.11
	POST	0.04	0.34	0.54	0.33±0.06	0.96±0.1	0.26±0.11	0.57±0.08	0.26±0.06	0.64±0.17
7	PRE	-0.06	0.26	0.14	0.71±0.06	1.06±0.03	0.45±0.12	0.94±0.12	0.98±0.06	0.91±0.05
	POST	0.26	0.24	0.68	0.34±0.11	0.87±0.03	0.27±0.07	0.69±0.03	0.26±0.05	0.94±0.11
8	PRE	-0.04	0.40	0.38	0.81±0.04	0.36±0.07	0.37±0.12	0.32±0.11	0.58±0.11	0.58±0.09
	POST	-0.06	0.58	0.56	0.82±0.05	0.19±0.06	0.74±0.05	0.47±0.12	0.26±0.12	0.45±0.1
9	PRE	0.26	0.22	0.32	0.86±0.04	0.55±0.04	0.7±0.11	0.71±0.09	0.55±0.06	0.65±0.08
	POST	0.16	0.68	0.44	0.65±0.1	0.2±0.03	0.43±0.16	0.19±0.2	0.56±0.13	0.61±0.15

Table 3: A summary of functional behavioral and physiological outcome data measured preoperatively (PRE) and 6 weeks postoperatively (POST). Dynamic Visual Acuity (DVA), Video Head Impulse Test (VHIT) mean and standard deviation, Anterior (Ant), Posterior (Post). *DVA is corrected to account for static visual acuity.
